# The Effects of Chronic Immunosuppressive Treatment on Morphological Changes in Cardiac Tissue and the Balance between Matrix Metalloproteinases (MMP-2 and MMP-9) and Their Inhibitors in the Rat Heart

**DOI:** 10.3390/ijms25084468

**Published:** 2024-04-18

**Authors:** Anna Surówka, Michał Żołnierczuk, Piotr Prowans, Marta Grabowska, Patrycja Kupnicka, Marta Markowska, Joanna Olejnik-Wojciechowska, Zbigniew Szlosser, Aleksandra Wilk, Kamila Szumilas, Karolina Kędzierska-Kapuza

**Affiliations:** 1 Department of Plastic, Endocrine and General Surgery, Pomeranian Medical University, 72-010 Szczecin, Poland; 2 Department of Vascular Surgery, General Surgery and Angiology, Pomeranian Medical University, 70-111 Szczecin, Poland; 3 Department of Histology and Developmental Biology, Faculty of Health Sciences, Pomeranian Medical University, 70-111 Szczecin, Poland; 4 Department of Biochemistry and Medical Chemistry, Pomeranian Medical University, Powstańców Wlkp. 72, 70-111 Szczecin, Poland; 5 Department of Plastic and Reconstructive Surgery, 109 Military Hospital, 71-422 Szczecin, Poland; 6 Department of Histology and Embryology, Pomeranian Medical University, 70-111 Szczecin, Poland; aleksandra.wilk@pum.edu.pl; 7 Department of Physiology, Pomeranian Medical University, 70-111 Szczecin, Poland; 8 Department of Gastroenterological Surgery and Transplantology, Centre of Postgraduate Medical Education in Warsaw, 137 Woloska St., 02-507 Warsaw, Poland

**Keywords:** immunosuppression, cyclosporine, tacrolimus, rapamycin, mycophenolate, matrix metalloproteinase, tissue inhibitor of matrix metalloproteinase

## Abstract

Using different three-drug immunosuppressive treatment regimens in a rat model, we aimed to determine the effects of long-term therapy on metalloproteinase-2 and metalloproteinase-9 activity and the expression of their inhibitors, as well as to assess the morphology of the animals’ cardiac tissue. Our results suggest that chronic use of immunosuppressive drugs disrupts the balance between the activity of MMPs and TIMPs. Depending on the type of drug regimen used, this leads to abnormalities in the cardiac structure, collagen fiber accumulation, or cardiomyocyte hypertrophy. The information obtained in the present study allows us to conclude that the chronic treatment of rats with the most common clinical immunosuppressive regimens may contribute to abnormalities in the myocardial structure and function. The results presented in this study may serve as a prelude to more in-depth analyses and additional research into the optimal selection of an immunosuppressive treatment with the lowest possible risk of cardiovascular complications for patients receiving organ transplants.

## 1. Introduction

Immunosuppressants are a group of drugs commonly used in organ transplant patients. They work by inhibiting the recipient’s immune response, preventing the acute rejection of the transplanted organ and preserving its normal function [1,2]. A standard treatment is based on the use of several drugs with different mechanisms of action at the lowest possible therapeutic doses. The most commonly chosen immunosuppressive regimen in renal transplant patients includes a calcineurin inhibitor (CNI), tacrolimus (TAC), cyclosporin A (CsA), mycophenolate mofetil (MMF), and corticosteroid (CS). This protocol results in one-year graft survival in 90% of cases and avoids episodes of acute organ rejection in 80% of cases. Alternative treatment regimens allow the use of an mTOR inhibitor, including rapamycin, as an alternative to CNI or MMF [3,4,5,6,7].

Unfortunately, despite their high efficacy in suppressing the immune response, immunosuppressive drugs are associated with numerous side effects. Among others, the use of these drugs increases the risk of infection and cancer and may exert toxic effects on parenchymal organs or the nervous system [8,9,10,11,12,13]. Currently, attention is focused on the increased risk of cardiovascular disease (CVD) in renal transplant recipients, as this is one of the leading causes of death in this patient population [14]. Patients with chronic renal failure are already burdened with factors that increase the risk of CVD prior to surgical treatment, such as diabetes, hypertension, dyslipidemia, or tobacco use [15,16]. The side effects of chronic immunosuppressive therapy promote pathological changes in the myocardial structure [17]. These drugs can affect tissue remodeling by disrupting the balance between metalloproteinases (MMPs) and tissue inhibitors of metalloproteinases (TIMPs). This leads to the remodeling of the extracellular matrix (ECM), cell hypertrophy, accumulation of collagen fibers, and ultimately limits the normal function of the entire organ [7,18]. 

### Metalloproteinases and Their Inhibitors in Heart Diseases

Extracellular matrix metalloproteinases (MMPs) are a group of more than a dozen endopeptidases with similar structural and enzymatic activities. Their purpose is to maintain tissue equilibrium during the degradation and synthesis of components of the extracellular matrix [19]. Abnormalities in the ECM structure are a substrate for progressive heart disease as well as a result of its inadequate remodeling, e.g., in heart failure, myocardial infarction, and ischemia. In the cardiovascular system, a special role is played by gelatinases, MMP-2 and MMP-9, which have the ability to hydrolyze type III collagen, type IV collagen, type V collagen, elastin, laminin, and fibronectin. They are secreted by cardiomyocytes, neutrophils, endothelial cells, macrophages, and fibroblasts [20,21,22,23]. 

MMP-2 is an independent predictor of cardiovascular prognosis and mortality [22,24]. An increased expression of MMP-9 is also correlated with the occurrence of CVD [23,25]. Specific inhibitors of MMP-2 and MMP-9 activity are TIMP-2 and TIMP-1, respectively. Under the influence of damaging factors such as hypoxia, increased oxidative stress, inflammation, and toxic effects of drugs, the balance between these two groups of proteins can be disturbed, which leads to the loss of tissue integrity, morphological and morphometric changes, and pathological neovascularization. A loss of control and imbalance between MMPs and TIMPs may contribute to complications such as myocardial infarction, atrial fibrillation, endocarditis and myocarditis, cardiomyopathy, and heart failure (Table 1) [26,27,28,29,30,31,32,33,34,35,36,37].

Immunosuppressive drugs may affect the MMP/TIMP imbalance in the heart, leading to connective tissue accumulation and myocardial fibrosis, degenerative changes in muscle fibers, and pathological tissue remodeling [7,38]. Currently, there is a lack of studies in the literature indicating the effects of commonly used triple-drug immunosuppressive treatment regimens on morphological changes in the heart and the activities of MMP-2, MMP-9, TIMP-1, and TIMP-2. Obtaining such knowledge is important in order to be able to monitor and eliminate certain factors that increase the risk of loss of normal cardiac function. The aim of this work is to test the effect of a triple immunosuppressive therapy on the morphological changes in myocardial tissue and the balance between matrix metalloproteinases (MMP-2 and MMP-9) and their inhibitors in the rat heart.

## 2. Results

### 2.1. Histological Analysis

**Figure 1:**
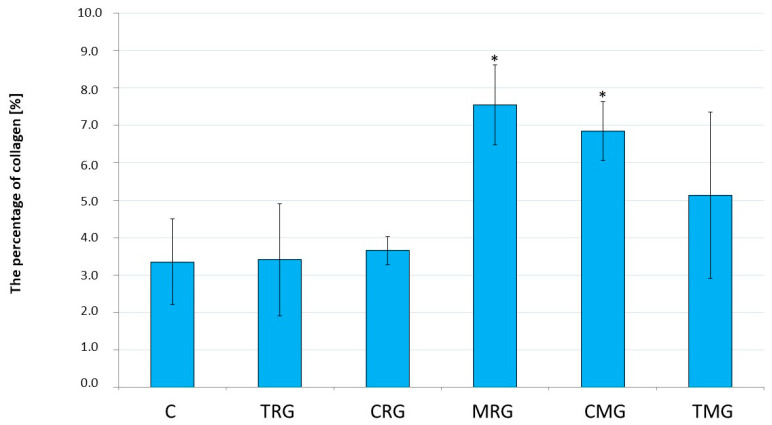
Comparison of the percentages of collagen in the hearts of rats between the control and experimental groups. Results are expressed as the mean and standard deviation. * *p* < 0.05 vs. control. C—control group without medication, (*n* = 6); TRG—rats treated with rapamycin, tacrolimus, and glucocorticosteroids, (*n* = 6); CRG—rats treated with rapamycin, cyclosporin A, and glucocorticosteroids, (*n* = 4); MRG—rats treated with rapamycin, mycophenolate mofetil, and glucocorticosteroids, (*n* = 6); CMG—rats treated with cyclosporin A, mycophenolate mofetil, and glucocorticosteroids, (*n* = 6); TMG—rats treated with tacrolimus, mycophenolate mofetil, and glucocorticosteroids, (*n* = 6).

In the rat hearts, the percentages of collagen obtained (*n* = 6 in each group except CRG group, in which *n* = 4) were significantly higher in the MRG (*p* = 0.009) and CMG (*p* = 0.039) groups than in the control group. In the TRG, CRG, and TMG groups, the above parameters were not statistically significant compared to the control group (Figure 1).

**Figure 2:**
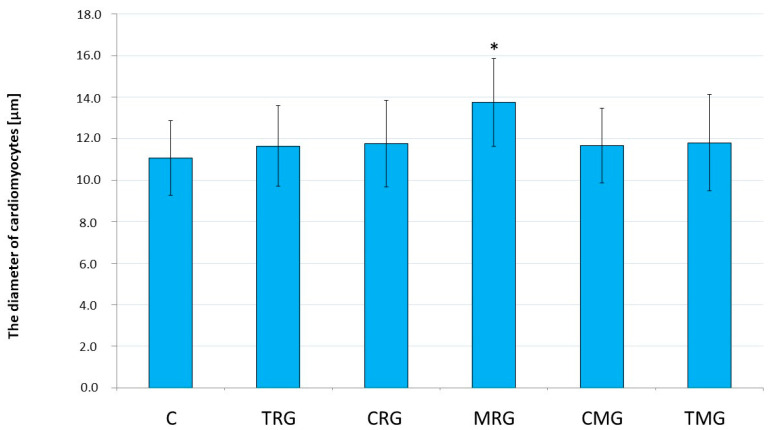
Comparison of cardiomyocyte diameters in the hearts of rats between control and experimental groups. Results are expressed as the mean and standard deviation. * *p* < 0.05 vs. control. C—control group without medication, (*n* = 6); TRG—rats treated with rapamycin, tacrolimus, and glucocorticosteroids, (*n* = 6); CRG—rats treated with rapamycin, cyclosporin A, and glucocorticosteroids, (*n*= 4); MRG—rats treated with rapamycin, mycophenolate mofetil, and glucocorticosteroids, (*n* = 6); CMG—rats treated with cyclosporin A, mycophenolate mofetil, and glucocorticosteroids, (*n* = 6); TMG—rats treated with tacrolimus, mycophenolate mofetil, and glucocorticosteroids, (*n* = 6).

The analysis of the rat hearts revealed mild cardiomyocyte hypertrophy in the MRG group. The cardiomyocyte diameter was significantly increased in the MRG group compared to the control group (*p* < 0.001). In the other groups, the above parameters were not statistically significant compared to control (Figure 2).

**Figure 3:**
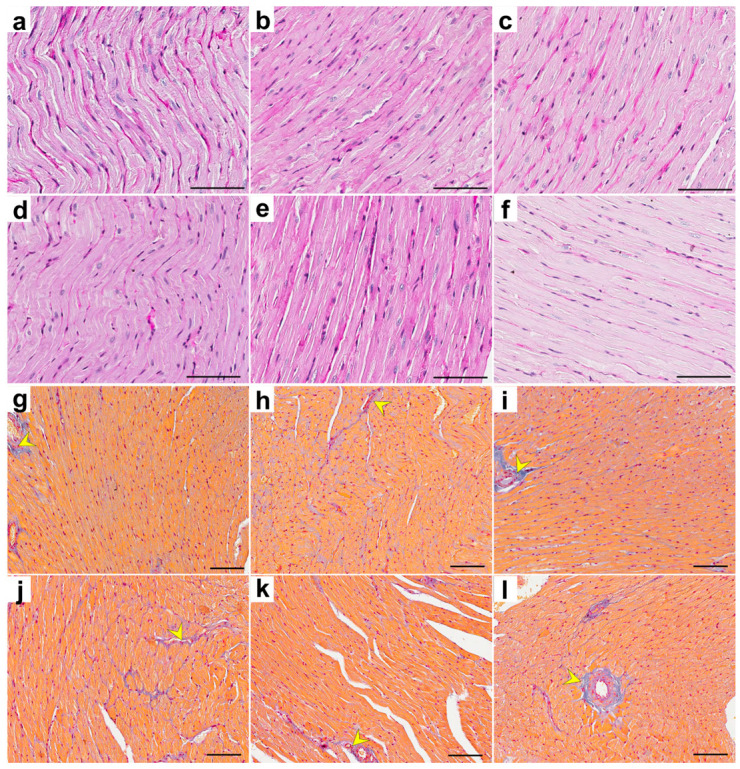
Representative photomicrographs of rat heart tissue stained with PAS and Masson’s trichrome. PAS-stained tissue in the control (**a**), TRG (**b**), CRG (**c**), MRG (**d**), CMG (**e**), and TMG (**f**) groups. Masson’s trichrome-stained collagen fibers (blue fibers and yellow arrowheads) were observed in the control (**g**), TRG (**h**), CRG (**i**), MRG (**j**), CMG (**k**), and TMG (**l**) groups. C—control group without medication, (*n* = 6); TRG—rats treated with rapamycin, tacrolimus, and glucocorticosteroids, (*n* = 6); CRG—rats treated with rapamycin, cyclosporin A, and glucocorticosteroids, (*n* = 4); MRG—rats treated with rapamycin, mycophenolate mofetil, and glucocorticosteroids, (*n* = 6); CMG—rats treated with cyclosporin A, mycophenolate mofetil, and glucocorticosteroids, (*n* = 6); TMG—rats treated with tacrolimus, mycophenolate mofetil, and glucocorticosteroids, (*n* = 6). PAS—periodic acid–Schiff. Scale bar—50 µm.

Representative photomicrographs of rat heart tissue stained with PAS (cardiomyocyte diameter) and Masson’s trichrome (analysis of collagen) are shown below (Figure 3).

### 2.2. Activities of MMP-2 and MMP-9 and Expressions of TIMP-1 and TIMP-2

#### 2.2.1. MMP-9 Activity (Zymography)

**Figure 4:**
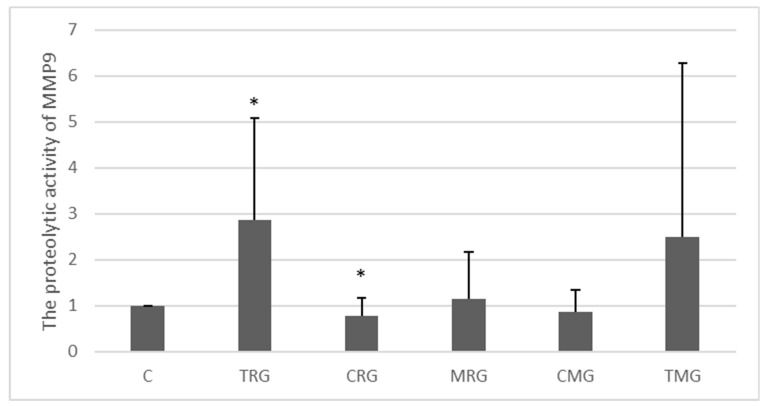
Semiquantitative densitometric analysis of matrix metalloproteinase-9 (MMP-9) activity in the hearts of rats from the C—control group without medication, (*n* = 6); TRG—rats treated with rapamycin, tacrolimus, and glucocorticosteroids, (*n* = 6); CRG—rats treated with rapamycin, cyclosporin A, and glucocorticosteroids, (*n* = 4); MRG—rats treated with rapamycin, mycophenolate mofetil, and glucocorticosteroids, (*n* = 6); CMG—rats treated with cyclosporin A, mycophenolate mofetil, and glucocorticosteroids, (*n* = 6); and TMG—rats treated with tacrolimus, mycophenolate mofetil, and glucocorticosteroids, (*n* = 6). MMP-9 was determined using zymography. MMP-9 observed molecular weight is 84–92 kDa. Data represent the mean ± standard deviation of six independent experiments. * *p* < 0.05 (Mann–Whitney U test).

The activity of MMP-9 in the examined tissues was generally low (Figure 4). However, in the TRG group, a 187% increase in the activity of MMP-9 was observed compared to the C group (*p* < 0.05). A significant difference was also observed in the CRG group, where the expression of MMP-9 was decreased by 22% compared to control (Figure 4).

#### 2.2.2. MMP-2 Activity (Zymography)

**Figure 5:**
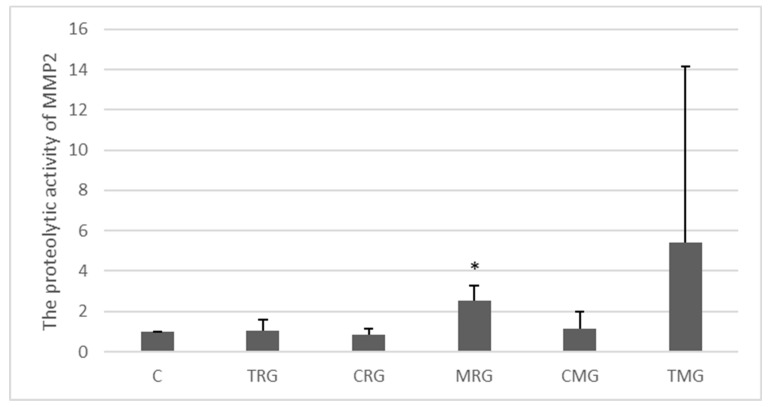
Semiquantitative densitometric analysis of matrix metalloproteinase-2 (MMP-2) activity in the hearts of rats from the C—control group without medication, (*n* = 6); TRG—rats treated with rapamycin, tacrolimus, and glucocorticosteroids, (*n* = 6); CRG—rats treated with rapamycin, cyclosporin A, and glucocorticosteroids, (*n* = 4); MRG—rats treated with rapamycin, mycophenolate mofetil, and glucocorticosteroids, (*n* = 6); CMG—rats treated with cyclosporin A, mycophenolate mofetil, and glucocorticosteroids, (*n* = 6); and TMG—rats treated with tacrolimus, mycophenolate mofetil, and glucocorticosteroids, (*n* = 6). MMP-2 was determined using zymography. MMP-2 observed molecular weight is 62–72 kDa. Data represent the mean ± standard deviation of six independent experiments. * *p* < 0.05 (Mann–Whitney U test).

The zymographic analysis showed that the level of MMP-2 activity in the tissues studied was generally low (Figure 5). The only significant change was observed in the MRG group vs. C group, where the activity of the MMP under study was 153% higher (*p* < 0.05). The highest average level of activity was observed in the TMG group, but the difference was not significant.

**Figure 6:**
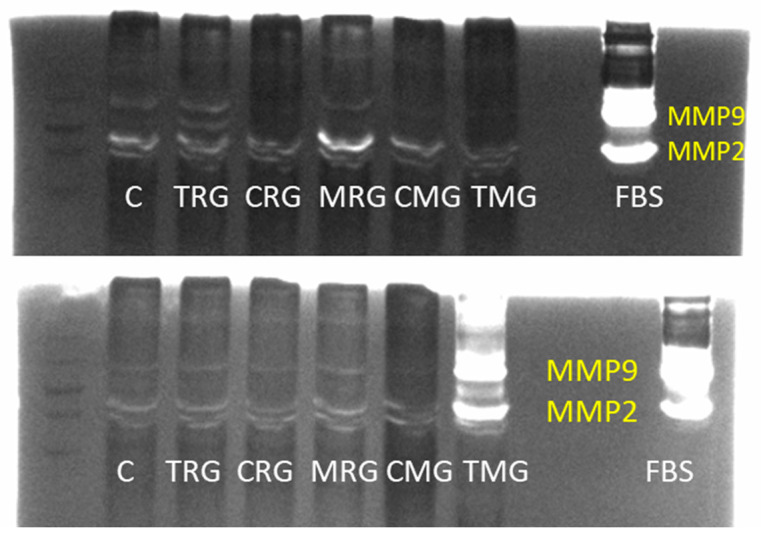
Visualization of gelatin zymography determining the levels of matrix metalloproteinase 2 (MMP2) and 9 (MMP9) in the hearts of the experimental groups and fetal bovine serum (FBS) as a positive control. C—control group without medication, (*n* = 6); TRG—rats treated with rapamycin, tacrolimus, and glucocorticosteroids, (*n* = 6); CRG—rats treated with rapamycin, cyclosporin A, and glucocorticosteroids, (*n* = 4); MRG—rats treated with rapamycin, mycophenolate mofetil, and glucocorticosteroids, (*n* = 6); CMG—rats treated with cyclosporin A, mycophenolate mofetil, and glucocorticosteroids, (*n* = 6); TMG—rats treated with tacrolimus, mycophenolate mofetil, and glucocorticosteroids, (*n* = 6). MMP-9 observed molecular weight is 84–92 kDa. MMP-2 observed molecular weight is 62–72 kDa.

Gelatin zymography visualization determining the levels of matrix metalloproteinase 2 (MMP2) and 9 (MMP9) in the hearts of the experimental groups and fetal bovine serum (FBS) as a positive control are presented in Figure 6.

#### 2.2.3. TIMP-1 Expression (Western Blot)

**Figure 7:**
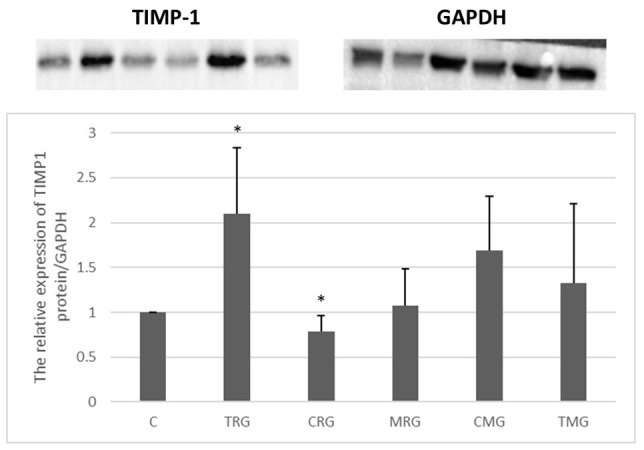
Representative blots and semiquantitative densitometric analysis of tissue inhibitor of metalloproteinases-1 (TIMP-1) protein expression in the hearts of rats from C—control group without medication, (*n* = 6); TRG—rats treated with rapamycin, tacrolimus, and glucocorticosteroids, (*n* = 6); CRG—rats treated with rapamycin, cyclosporin A, and glucocorticosteroids, (*n* = 4); MRG—rats treated with rapamycin, mycophenolate mofetil, and glucocorticosteroids, (*n* = 6); CMG—rats treated with cyclosporin A, mycophenolate mofetil, and glucocorticosteroids, (*n* = 6); and TMG—rats treated with tacrolimus, mycophenolate mofetil, and glucocorticosteroids, (*n* = 6). GAPDH - glyceraldehyde-3-phosphate dehydrogenase; TIMP-1 expression was determined using a Western blot analysis. Reference protein was GAPDH. Data represent the mean ± standard deviation of six independent experiments. * *p* < 0.05 (Mann–Whitney U test).

Each group, except the CRG group, was characterized by increased TIMP-1 protein expression compared to the control, but the change was significant only in the TRG group. In this group, the protein expression of TIMP-1 was more than 2-fold higher than in the control group (C) (*p* < 0.05). The lowest expression was observed in the CRG group, where the level of TIMP-1 protein was 21% lower than in the C group (*p* < 0.05) (Figure 7).

#### 2.2.4. TIMP-2 Expression (Western Blot)

**Figure 8:**
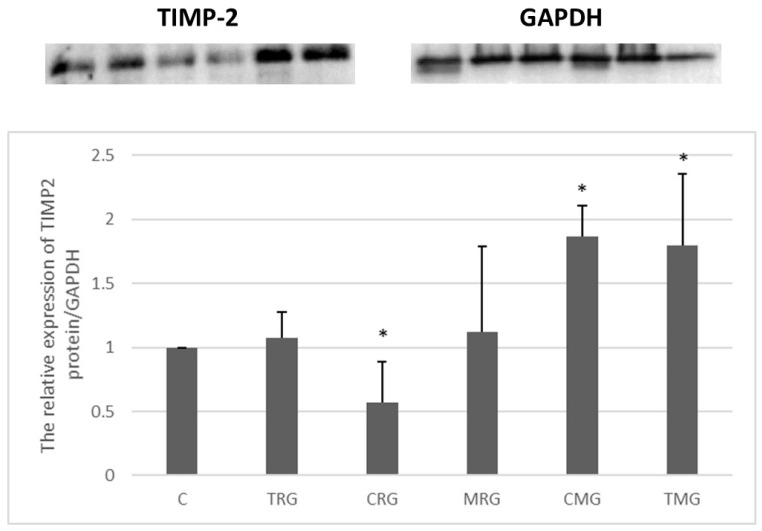
Representative blots and semiquantitative densitometric analysis of tissue inhibitor of metalloproteinases-2 (TIMP-2) protein expression in the hearts of rats from C—control group without medication, (*n* = 6); TRG—rats treated with rapamycin, tacrolimus, and glucocorticosteroids, (*n* = 6); CRG—rats treated with rapamycin, cyclosporin A, and glucocorticosteroids, (*n* = 4); MRG—rats treated with rapamycin, mycophenolate mofetil, and glucocorticosteroids, (*n* = 6); CMG—rats treated with cyclosporin A, mycophenolate mofetil, and glucocorticosteroids, (*n* = 6); and TMG—rats treated with tacrolimus, mycophenolate mofetil, and glucocorticosteroids, (*n* = 6). GAPDH - glyceraldehyde-3-phosphate dehydrogenase; TIMP-2 expression was determined using a Western blot analysis. Reference protein was GAPDH. Data represent the mean ± standard deviation of six independent experiments. * *p* < 0.05 (Mann–Whitney U test).

The CRG group was characterized by a 43% decrease in the expression of the TIMP-2 protein compared to the control (C) group (*p* < 0.05) (Figure 8). An increase in the expression was observed in the other groups, but it was significant only in the CMG and TMG groups (vs. C). The level of TIMP-2 expression was 87% higher in the CMG group and 80% higher in the TMG group (*p* < 0.05) (Figure 8).

## 3. Discussion

The aim of the experiment was to determine whether there are changes in the activities of MMP-2 and MMP-9 and in the expressions of TIMP-1 and TIMP-2 and whether there are apparent morphological abnormalities in rat cardiac tissue in the course of chronic administration of immunosuppressive drugs according to standard three-drug regimens. Tissue sections from the hearts of rats in the control and five experimental groups were analyzed in detail. The available literature contains descriptions of cardiovascular changes after administration of a single immunosuppressive drug, often at different doses, making it difficult to compare the results obtained with other data. 

The main factor in the development of heart failure (HF) is myocardial hypertrophy, including left ventricular hypertrophy (LVH), which is a compensatory response of the cardiovascular system to prolonged stressors such as hypertension, dyslipidemia, diabetes, or certain medications [39,40,41,42]. Under physiological conditions, cardiomyocytes represent approximately one-third of all cardiac cells and three-quarters of the mass of the entire organ. Cardiac cells are fully differentiated and do not have the ability to divide. Myocardial hypertrophy occurs through ECM remodeling, cellular hypertrophy, collagen fiber accumulation, and apoptosis. Advanced lesions are associated with increased cardiovascular risk, the occurrence of arrhythmias, myocardial diastolic dysfunction, and progression to full-blown heart failure [36,37,38].

Under physiological conditions, cells control the secretion of MMPs and TIMPs at a constant level. This allows for a balance between the degradation and synthesis of extracellular matrix elements [43,44]. Under the influence of stress factors, there is an increased synthesis of cytokines, growth factors, tumor necrosis factors, or free radicals, which stimulate an increase in the expression of metalloproteinases [45,46,47,48]. A gradual decrease in the activities of proteolytic enzymes or a compensatory increase in the expressions of inhibitors of metalloproteinases can lead to the excessive accumulation of collagen fibers or cellular hypertrophy [18,49].

In our study, we highlighted the levels of MMP-2 and MMP-9 activity and TIMP-1 and TIMP-2 expression in the cardiac tissues of rats receiving an immunosuppressive treatment for a period of six months, corresponding to approximately 15 years of therapy in organ transplant patients. The staining of the sections allowed for the visualization of cardiomyocytes and collagen fibers. In routinely stained slides, all study groups showed an increase in the cardiomyocyte diameter compared to the control group; however, only the mycophenolate–rapamycin–glucocorticoid (MRG) group showed statistically significant cell hypertrophy. In addition, an increase in the amount of collagen fibers in the cardiac tissues of rats treated with the above-mentioned three-drug regimen was demonstrated. These changes were accompanied by increased MMP-2 activity. 

According to the available literature, rapamycin may significantly affect the development of hypertension. This effect is related to both an increase in systolic and diastolic blood pressure and the number of heart beats per minute. In addition, the use of an mTOR inhibitor is associated with adverse effects on lipid metabolism, raising the blood levels of triglycerides and LDL cholesterol. These changes may increase the risk of cardiovascular events. A study by Cheng Long et al. [50] confirmed that rapamycin therapy significantly increased blood pressure in rats. Reduced NO activity in the vascular endothelium was demonstrated, resulting in impaired aortic relaxation. Cardiac stress in the mechanism of hypertension can lead to myocardial fibrosis due to the excessive deposition of extracellular matrix elements and cardiomyocyte hypertrophy. Researchers have suggested that an increase in MMP activity provides a cardioprotective mechanism by increasing the degradation of accumulated ECM proteins or altering collagen cross-linking. Blocking the activities of MMPs promotes cardiomyocyte hypertrophy, so metalloproteinases may play an important role in inhibiting cardiac hypertrophy [18]. The results obtained in our study may suggest an adverse effect of rapamycin on the cardiovascular system in the form of increased arterial pressure and its sequelae in the form of changes in the morphological structure of the heart. Increased MMP-2 activity may be a response to excessive collagen accumulation and an attempt to restore normal tissue homeostasis.

Numerous reports show that the incidence of adverse cardiovascular effects is greater with cyclosporin than with tacrolimus. Cyclosporin is more conducive to the development of hypertension, dyslipidemia, and increased body weight. The main known factor in the development of heart failure is chronic hypertension, which causes cellular hypertrophy, fibroblast influx, extracellular matrix remodeling, fibrosis and cardiomyocyte death. The most frequently cited cause of hypertension in organ recipients is chronic cyclosporin treatment. Clinically, the use of CsA is associated with a significantly higher risk of fibrosis and myocardial remodeling than TAC [7,17,43]. This phenomenon can also be seen in the results presented above. A cyclosporine–mycophentolate–glucocorticoid (CMG) triple therapy was shown to contribute to the increased deposition of collagen fibers in the heart, which was not seen in the experimental groups treated with tacrolimus-based drug regimens. In addition, the CMG and tacrolimus–mycophenolate–glucocorticoid (TMG) groups showed an increased expression of TIMP-2, which may significantly affect collagen fiber deposition by inhibiting MMP-2 activity and thus reducing protein degradation within the ECM. The TMG group showed an increase in collagen fibers. However, this was not statistically significant.

Bianchi et al. [51] investigated MMP-2 and vascular endothelial growth factor (VEGF) expression and morphological changes in the rat heart after 21 days of CsA treatment at a dose of 15 mg/kg/d. The animals in the test group showed features of myocardial fibrosis and a significant increase in both VEGF and MMP-2. The researchers attributed the role of the proteins to repair mechanisms against changes induced by stress factors: in this case, the administration of a cardiotoxic immunosuppressive drug. In our study, the rats treated with the CMG treatment regimen showed an increase in TIMP-2 expression without changes in MMP-2 and MMP-9 activity. It should be noted that the dose of CsA we used was three times lower and the duration of use was much longer than in the study by Bianchi et al. [51]. It is conceivable that the longer duration of drug exposure may have favored the compensatory mechanisms activated to protect the organ, which may have involved a decrease in the expressions of MMPs or the inhibition of their activities by TIMPs and other inhibitors. Cardiac remodeling induced by factors such as pressure overload, volume overload, or myocardial injury is a dynamic process consisting of several sequential stages. The effect of the injurious stimulus triggers an initial phase characterized by increased MMP activity and extracellular matrix remodeling. In the compensated phase, MMP activity is compensated, while progressive cardiac hypertrophy is observed. In the decompensated phase, there is a further increase in MMP activity, which no longer compensates for the progressive interstitial fibrosis process [52].

In another study, Berthier et al. [53] compared cardiac tissue changes in rats that underwent cardiac allograft transplantation without subsequent immunosuppressive treatment with those that received CsA after the procedure. The duration of drug administration was 6 days. In experimental acute allograft rejection, the mRNA levels of MMP-2, MMP-9, and TIMP-1 were increased, whereas TIMP-2 mRNA was decreased. Compared to untreated rats, CsA decreased TIMP-1 mRNA and increased the total MMP activity in both groups. Cyclosporin improved the rejection histology; however, it had profibrotic effects at the level of increased gene expression for ECM components such as collagen and laminin. It should be noted that the observed changes relate to the immediate post-transplant period and may be related to a compensatory increase in MMPs in response to direct myocardial damaging agents. However, this study confirms our results, demonstrating an effect of cyclosporin on excessive collagen fiber accumulation.

Mycophenolate mofetil, as an antimetabolic drug, may reduce fibrotic processes and have a protective effect on the cardiovascular system, thereby reducing the negative effects of calcineurin inhibitors. In the results presented here, significant morphological changes in rat cardiac tissue were observed only in the CMG group. It is plausible that in the treatment regimens used, cyclosporin, as opposed to tacrolimus, may have abolished the protective effect of mycophenolate mofetil to a greater extent. Similar findings were reported in a study by Kobashigawa et al. [54], which showed that treatment of heart transplant patients with TAC + MMF was more beneficial than the use of CsA + MMF. The rate of graft rejection and the number of side effects were lower in the tacrolimus group. Patients receiving tacrolimus had a worse lipid profile, higher serum creatinine levels, and a greater need for antihypertensive medication.

In our study, there was an increase in MMP-9 activity with a concomitant increase in TIMP-1 expression in the TRG group. In the cyclosporin–rapamycin–glucocorticoid (CRG) group, there was a decrease in MMP-9 activity and TIMP-1 expression. There were no statistically significant morphological changes in rat heart tissue in either study group. The results suggest that as the endopeptidase activity increased/decreased, there were corresponding changes in the expression of its inhibitors. The available literature reports that the combination of a calcineurin inhibitor with an mTOR inhibitor at lower doses has a more favorable effect than the use of cyclosporin at higher doses or as monotherapy. Brook et al., (2005) [55] found that the corresponding lower dose combination of cyclosporin (7.5 mg/kg/d) + rapamycin (0.5 mg/kg/d) tested in a rat model reduced side effects, including antiproliferative/antifibrotic effects within the kidney. This was due to the increased expression of MMP-2 and decreased expression of TIMP-1. In our model, a lower dose of cyclosporin (5 mg/kg/d) was used, but like Brook et al. [55], we found decreased expressions of both TIMPs tested, which may have promoted the maintenance of normal amounts of collagen fibers in rat heart tissue.

Tacrolimus has similar pharmacological properties to cyclosporine. Our results may suggest that the combination of an mTOR inhibitor with a calcineurin inhibitor may have been more beneficial than the use of these drugs in separate regimens. 

The information obtained in the present study allows us to conclude that the chronic treatment of rats with the most common clinical immunosuppressive regimens may contribute to abnormalities in the myocardial structure and function. We observed changes in the activities of MMPs or in the expressions of TIMPs in each study group. Furthermore, regimens based on cyclosporin and rapamycin in combination with mycophenolate mofetil showed the greatest effect on ECM remodeling. Only rapamycin affected cardiomyocyte hypertrophy. Both drugs abolished the protective effect of MMF. The combination of a calcineurin inhibitor with an mTOR inhibitor did not affect the cardiac morphological parameters studied, and changes in MMP activity were compensated by changes in TIMP expression. In contrast to cyclosporine, tacrolimus showed less fibrotic potential. The results obtained may be a prelude to deeper analyses and additional studies on the optimal selection of an immunosuppressive treatment with the lowest risk of cardiovascular complications for patients undergoing organ transplantation.

## 4. Material and Methods

### 4.1. Animals and Protocol

**Figure 9:**
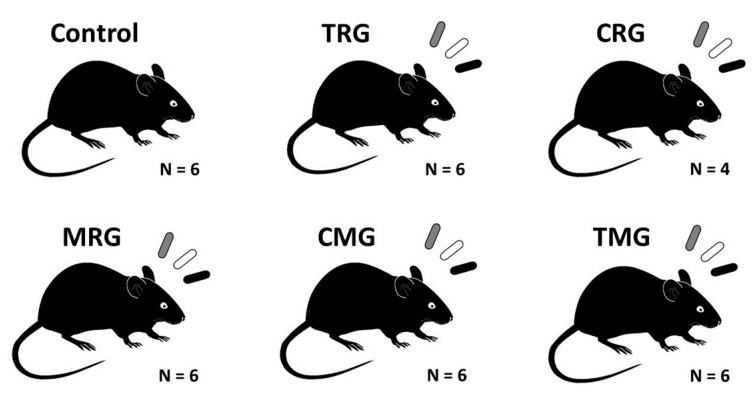
Groups of Wistar rats used in the experiment. Control—control group without any medication; TRG—rats treated with rapamycin, tacrolimus, and glucocorticosteroids; CRG—rats treated with rapamycin, cyclosporin A, and glucocorticosteroids; MRG—rats treated with rapamycin, mycophenolate mofetil, and glucocorticosteroids; CMG—rats treated with cyclosporin A, mycophenolate mofetil, and glucocorticosteroids; TMG—rats treated with tacrolimus, mycophenolate mofetil, and glucocorticosteroids.

Thirty-six Wistar rats aged 14 weeks were included in the study. All animals were male, and the average body weight was 305 g. The experiment was approved by the Local Ethical Committee for Animal Experiments in Szczecin. In the animal room, the air temperature was 22 ± 2 °C. The air humidity was 55%. The 12/12 light day was regulated automatically. Rats were fed a specialized laboratory diet LSM (1476 kJ/100 g, 17.6% protein). Water was provided for drinking. After a 2-week adaptation period, the rats were divided into 6 groups, with 6 animals in each group. The animals in each group received combinations of drugs corresponding to the most commonly used three-drug immunosuppressive regimens (Figure 9). The control group did not receive any drugs.

Pharmaceutical dosage forms were used in the experiment. The doses were adjusted based on the available literature data. The drugs were administered to the rats orally in the form of a bread ball once a day for a period of 6 months. Halfway through the experiment, the animals were weighed again, and the drug dose was adjusted according to the weight (Table 2).

Two animals in the CRG group died in the middle of month 4. After 6 months of the experiment, the animals were anesthetized by intraperitoneal administration of ketamine and then euthanized. The animals were necropsied, and tissue sections of the hearts were collected. Some of the material was placed in a vat of liquid nitrogen and then preserved in a low-temperature freezer (−86 °C). The remaining heart sections were fixed in 4% paraformaldehyde and embedded in paraffin blocks.

### 4.2. Histological Analysis

Rat hearts were fixed in 4% buffered formaldehyde, dehydrated through a graded series of ethyl alcohol and xylene, and embedded in paraffin. Paraffin blocks were cut into 3 μm thick sections. For staining using standard methods, the sections were deparaffinized in xylene and rehydrated in a graded ethyl alcohol series. The sections were stained with hematoxylin and eosin (H–E) and periodic acid–Schiff (PAS) to examine the general architecture of the cardiac tissue and with Mallory trichrome stain to visualize collagen fibers.

### 4.3. Quantitative Analysis of Cardiomyocyte Diameter and Mallory Trichrome Staining

H–E and Mallory trichrome stained slides were scanned at 400× magnification using a ScanScope AT2 scanner (Leica Microsystems, Wetzlar, Germany). The resulting digital images of the sections were then analyzed using ImageScope viewer software (Aperio Technologies, Vista, CA, USA).

The cardiomyocyte diameter (µm) was examined using a ruler tool on the PAS-stained sections. In each group, one hundred and thirty-eight longitudinally sectioned cells in the nuclear region were analyzed in each group (twenty-three cells in each rat).

A quantitative analysis of collagen was performed on Mallory’s trichrome-stained cardiac tissue sections. A positive pixel count v9 algorithm (Aperio Technologies, Inc., Vista, CA, USA) was used for this purpose. The areas of analysis were determined manually. The percentage of collagen fibers (positive for Mallory’s trichrome staining) was determined in 36 random high-power fields (6 for each group).

### 4.4. Homogenization of Samples and Determination of Protein Concentration

Frozen heart samples were transferred individually to a metal homogenizer cooled with liquid nitrogen. The tissues were comminuted by striking a cooled metal mandrel with a hammer. After each use, the equipment was cleaned and cooled in liquid nitrogen. The prepared samples were treated with RIPA Lysis Buffer (Cat. 89901, Thermo Scientific, Pierce Biotechnology, Waltham, MA, USA). The samples were mixed thoroughly and incubated on ice for 20 min, followed by centrifugation (20 min, 14,000 rpm, 4 °C). The protein-containing supernatant was transferred to new Eppendorf tubes. The total protein concentration was determined using the MicroBCA Protein Assay Kit (cat. 23235, Thermo Scientific, Pierce Biotechnology, Waltham, MA, USA) according to the manufacturer’s instructions. The absorbance of the samples was determined using a Biochrom EZ Read 2000 plate reader (wavelength 562 nm). The protein concentration was calculated from a simultaneously generated standard curve.

### 4.5. Activities of MMP-2 and MMP-9 

For the analysis of MMP-2 and MMP-9 activity, 7.5% acrylamide and 1% gelatin gels were prepared. Each sample contained 80 µg protein. Laemmli buffer (Laemmli Sample Buffer, Bio-Rad, Feldkirchen, Germany) was added to the samples. The final sample volume was 15 µL. The samples were loaded onto the prepared gels and placed in an electrophoresis chamber filled with electrode buffer (25 mM Tris/250 mM glycine/0.1% SDS; Merck, Germany). A mass standard was applied to the gel: BlueAQUA Prestained Protein Ladder (GeneDireX Inc., Taoyuan, Taiwan) and a standard containing the tested metalloproteinases (Bovine Serum Albumin, Biowest, France) together with the test samples. The electrophoresis was performed under the voltage of 125 V for 90 min at a temperature of 4 °C. After electrophoresis, the gels were incubated in a renaturation buffer (2.5% Triton X-100, Thermo Fisher Scientific, Waltham, MA, USA) at room temperature to elute with SDS and restore the enzymatic activity. After removing the renaturation buffer, the gels were rinsed with distilled water and then incubated in a buffer containing the cofactors necessary for the gelatin hydrolysis reaction with the MMPs under study (50 mM Tris, 10 mM CaCl_2_, 0.02% NaN_3_, pH = 7.4, Sigma-Aldrich, St. Louis, MO, USA). An incubation was performed at 37 °C for 48 h. The gels were stained with Coomassie Blue solution (Coomassie Brilliant Blue R-250, Thermo Fisher Scientific, Waltham, MA, USA). The gels were then washed several times with a destaining solution (10% methanol, 5% acetic acid; Merck, Germany) until clear bands were obtained. The gels were photographed using a transilluminator (Molecular Imager ChemiDock XRS +; Bio-Rad, Hercules, CA, USA). A densitometric analysis was performed using Image Lab software 6.1.0 (Bio-Rad Laboratories, Inc., Hercules, CA, USA).

### 4.6. Expressions of TIMP-1 and TIMP-2

Electrophoretic protein fractionation was performed on a 14% polyacrylamide gel at 30 μg protein/well. Prior to electrophoresis, the samples were heated at 90 °C for 7 min in 2-mercaptoethanol (Sigma-Aldrich, St. Louis, MO, USA) and Laemmli buffer (Laemmli Sample Buffer, Bio-Rad, Feldkirchen, Germany). The fractionated proteins were transferred using wet transfer to a 0.2 μm PVDF membrane (Thermo Fisher Scientific™, Waltham, MA, USA). Prior to incubation with antibodies, the membranes were placed in a blocking buffer (5% BSA) for 60 min. Protein expression was detected using antibodies against TIMP-1 (sc21734) and TIMP-2 (sc71735) (Santa Cruz Biotechnology, Dallas, TX, USA) diluted 1:100, and sAb goat anti-mouse IgG HRP H&L (ab6789) (Abcam, Cambridge, UK). The expression of the reference protein GAPDH (ab8245) was detected. The membranes were developed using an ECL Advance Western Blotting Detection Kit (GE Healthcare, Chicago, IL, USA), and the bands were visualized using a ChemiDock XRS+ molecular imager (Bio-Rad, Hercules, CA, USA).

### 4.7. Statistical Analysis

The results were statistically analyzed using Statistica 13.1 software; they are presented as the mean ± SD. The Shapiro–Wilk W test did not show agreement with a normal distribution; the non-parametric Mann–Whitney U test was used to compare groups. Statistical significance was set at *p* < 0.05.

## Figures and Tables

**Table 1:** Changes in MMP and TIMP serum levels and expression in various cardiovascular disease entities.

Disease Entity	MMP	Effect	Reference
Myocardial infarction	MMP-1 TIMP-1	Increased serum MMP1 and TIMP-1 levels in post-MI patients undergoing coronary artery reperfusion	[26]
	MMP-9	Increased serum MMP-9 levels as a predictive factor in patients with coronary artery disease	[27]
	MMP-2 MMP-9	Increased serum levels of MMP-2 and MMP-9 in patients with myocardial ischemia	[28]
	MMP-2	Increased tissue expression of MMP-2 in rat hearts after reperfusion following myocardial ischemia	[29]
Atrial fibrillation	MMP-9	Increased tissue expression of the active form of MMP-9 in patients with atrial fibrillation compared with controls without a history of AF	[30]
	MMP-9	Increased serum levels of the active form of MMP-9 in patients with atrial fibrillation compared with controls without a history of AF	[31]
Endocarditis	MMP-9	Increased serum MMP-9 levels in patients with an embolic episode in infective endocarditis	[32]
Cardiomyopathy	MMP-2 MMP-9	Increased tissue expressions of MMP-2 and MMP-9 in hearts of rats with inflammation and fibrosis compared to non-diseased hearts of healthy rats	[33]
Heart failure	MMP-2 MMP-9 TIMP-1	Increased serum levels of MMP-2 and MMP-9 and TIMP-1 in patients with heart failure compared to controls	[34]
	MMP-1 TIMP-1	Higher serum TIMP-1 and lower serum MMP-1 levels in patients with heart failure; TIMP-1 levels and the TIMP-1/MMP-1 ratio correlate negatively with peak VO2	[35]
Myocarditis	MMP-2 MMP-9	Increased tissue expressions of MMP-2 and MMP-9 in Coxsackie B3 virus-induced myocarditis in mice	[36]
	MMP-2	Negative correlation of cardiac ejection fraction and serum MMP-2 levels in patients with myocarditis	[37]

**Table 2:** Immunosuppressive drug doses used in the experiment.

Drug Name	Dose	Trade Name
Cyclosporin A	5 mg/kg/day	Sandimmun-Neoral
Tacrolimus	4 mg/kg/day	Prograf
Mycophenolate mofetil	20 mg/kg/day	Cellcept
Rapamycin	0.5 mg/kg/day	Rapamune
Prednisone	4 mg/kg/day	Encorton

## Data Availability

The data presented in this study are available upon reasonable request from the corresponding author.
